# Impact of CMOS Pixel and Electronic Circuitry in the Performance of a Hartmann-Shack Wavefront Sensor

**DOI:** 10.3390/s18103282

**Published:** 2018-09-29

**Authors:** Úrsula Vasconcelos Abecassis, Davies William de Lima Monteiro, Luciana Pedrosa Salles, Carlos Augusto de Moraes Cruz, Pablo Nunes Agra Belmonte

**Affiliations:** 1 Department of Electronics and Telecommunications, Instituto Federal do Amazonas, Av. Governador Danilo Areosa, 1672—Distrito Industrial, Manaus-AM 69075-351, Brazil; 2 Department of Electrical Engineering, DEE/PPGEE, Universidade Federal de Minas Gerais, Av. Antonio Carlos, 6627—Pampulha, Belo Horizonte-MG 31270-010, Brazil; davies@ufmg.br (D.W.d.L.M.); pablonuneagb@gmail.com (P.N.A.B.); 3 Department of Electronics and Computation, Universidade Federal do Amazonas, Manaus-AM 69077-000, Brazil

**Keywords:** wavefront sensor, adaptive optics, SPICE, CMOS, simulation, active pixel, quad-cell, integrated circuits

## Abstract

This work presents a numerical simulation of a Hartmann-Shack wavefront sensor (WFS) that assesses the impact of integrated electronic circuitry on the sensor performance, by evaluating a full detection chain encompassing wavefront sampling, photodetection, electronic circuitry and wavefront reconstruction. This platform links dedicated C algorithms for WFS to a SPICE circuit simulator for integrated electronics. The complete codes can be easily replaced in order to represent different detection or reconstruction methods, while the circuit simulator employs reliable models of either off-the-shelf circuit components or custom integrated circuit modules. The most relevant role of this platform is to enable the evaluation of the applicability and constraints of the focal plane of a given wavefront sensor prior to the actual fabrication of the detector chip. In this paper, we will present the simulation results for a Hartmann-Shack wavefront sensor with an orthogonal array of quad-cells (QC) integrated along with active-pixel (active-pixel sensor (APS)) circuitry and analog-to-digital converters (ADC) on a “complementary metal oxide semiconductor” (CMOS) process and deploying a modal wavefront reconstructor. This extended simulation capability for wavefront sensors enables the test and verification of different photosensitive and circuitry topologies for position-sensitive detectors combined with the simulation of sampling microlenses and reconstruction algorithms, with the goal of enhancing the accuracy in the prediction of the wavefront-sensor performance before a detector CMOS chip is actually fabricated.

## 1. Introduction

Adaptive optics (AO) has emerged as a technology focused on improving the performance of optical systems, by reducing the deleterious effects caused by wavefront (WF) distortions. Adaptive optical systems (AOS) usually use several systems and components for the detection and dynamic compensation of aberrations, such as deformable mirrors, lasers, precision optical parts and wavefront sensors (WFS) [1]. The applications of this technology can be found in multiple fields, as ophthalmology [2], industry [3] and astronomy [4], each with their own particularities. It is, therefore, useful to have a computational platform that models a wavefront sensor (WFS) assisting in the design and optimization of the system in order to better suit a specific application [5,6]. There have been several adaptive optical system simulation proposals implemented and made openly available in recent years, mostly meant for astronomy, but with a potential for deployment in other fields, as well [7,8,9,10,11,12,13]. In this paper, a Hartmann-Shack WFS will be modeled with an orthogonal array of quad-cells integrated along with CMOS active-pixel integrated circuitry and with a modal wavefront-reconstruction algorithm.

The simulation contains every step in the WF detection and reconstruction process, from the generation of the input WFs to the computation of the reconstructed WFs and their respective resulting errors. It guides the designer through the evaluation of different WF sensing techniques and structures that composes a Hartmann-Shack WFS prior to device fabrication, as the scheme for the pixel read-out circuitry, as well as the topology of the microlens array. The Hartmann-Shack detection method was chosen since it is the most ubiquitously-used WF detection method [14,15]. The introduction of integrated-circuit device models allows a more accurate prediction of the sensor performance. This was done by incorporating a simulation program with integrated circuit emphasis (SPICE), a reliable circuit simulator that emulates the behavior of semiconductor electronic devices, using verified and updated semi-empirical device models from a commercial semiconductor foundry.

A WF is represented as a smooth and well-behaved two-dimensional mathematical function, whose optimal shape depends on the target application. Deviations from its optimal shape are considered as optical aberrations. Traditionally, methods for WF reconstruction are classified into zonal estimates [16], where discrete wavefront patches are titled together, and modal estimates [17], where a weighted sum of continuous mathematical base functions represents the WF, aberrated or not. The modal reconstruction is widely used in adaptive optics [18,19], and an appropriate set of base functions are the Zernike polynomials [20], which will be employed in this paper.

**Figure 1:**
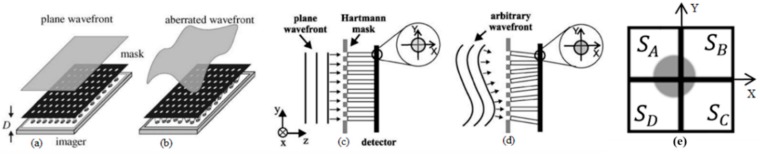
Hartmann-Shack sensor with the incidence of a wavefront (WF) plane and aberrated WF [24]. (**a**) WF focusing on the optical detection plane resulting spots located in the center of each opening of the microlens; (**b**) WF with aberration focusing on the optical detection plane resulting in spots shifted from its reference position; (**c**) side view of (a) highlighting the location of the spot in the detection plane; (**d**) side view of (b) highlighting the spot location diverted from its reference; (**e**) spot on the surface of a QC.

The aforementioned Hartmann-Shack WFS is mainly composed of a microlens array and an optical detection plane, located at the focal plane of the microlens array. The latter is meant to sample and focus patches of the incident WF onto the detection plane, resulting in one light spot for each microlens on the sampling plane. The spot positions depend on the average local tilts of the sampled WF. If the WF is parallel to the microlens array (Figure 1a), the spots then lie precisely under the center of their respective microlenses (Figure 1c), representing the vertices of a reference spot grid. Otherwise, for an arbitrary WF (Figure 1b), the spots will be displaced from that grid (Figure 1d). The dynamic detection of that displacement can be accomplished with either an image sensor or a dedicated array of position-sensitive detectors (PSDs). Each PSD is responsible for sensing the centroid of the focused light spot as electrical signals that can be then translated into x and y position coordinates. The position data from all PSDs and the distance between the detection plane and the Hartmann mask are used to reconstruct the wavefront [15].

In this work, PSDs of the quad-cell (QC) type are used [21]. A QC is composed by a quadrant, 2 × 2 array of independent photodetectors, as shown in Figure 1e, whose output signals are proportional to the light intensity on each of them, which in turn depends on the position of the light spot over the QC. In order to read its signal, each QC photodetector needs a read-out circuit. The smallest physical detection element of a WFS focal plane is a pixel, which comprises the photodetector and its read-out electronics. The most common and well-known type of pixel is called the active-pixel sensor (APS) [22], which is often fabricated in a standard CMOS process [23]. Details of the APS used are presented in Section 2.

In this work, simulations and their interpretation will be presented in the context of ophthalmology. However, it is worth mentioning that the simulation can be easily adapted to any other application in AO, provided that reference WF statistics or databases are available.

## 2. Simulation Method and Structure

**Figure 2:**
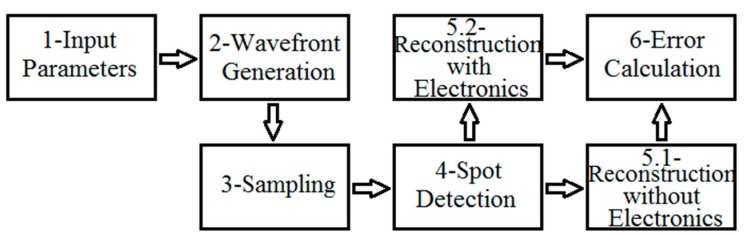
Block diagram of the simulation flow.

The computational structure to model the WF sensing flow is divided into six main blocks, as shown in Figure 2.

The parameters used to initialize the simulation in Step 1 of Figure 2 are the operational wavelength, the number of Zernike polynomial terms to be used for the WF generation, the sampling-related parameters, photodetection and the electronic circuitry (see Table A1 in Appendix A). In Step 2, the input WF is generated by a weighted sum of Zernike terms, whose coefficients ($C_{in}$) are based on aberration statistics of a population of human eyes, by Porter et al. [25]. Next, in Step 3, surface patches of the input WF are sampled by an orthogonal microlens array, whose structural characteristics are listed in Table A1. Then, the C algorithm analyses the sampled WF, translating the average tilt of each WF surface patch into the resulting x and y light-spot coordinates on its respective QC (Figure 1e), respectively XQC and YQC, relative to the QC center, for a given distance between the sampling and the detector planes.

The amount of light over a single photodetector is translated as a proportional photocurrent signal. Any deviation from the QC center renders different signal values for each photodetector, according to the model presented in [21]. Two kinds of quad-cells have been evaluated: the homogeneous (QC_H_), featuring homogeneous quantum efficiency on its surface; and the double quantum efficiency (QC_D_), which has two concentric regions with different quantum efficiencies [26].

**Figure 3:**
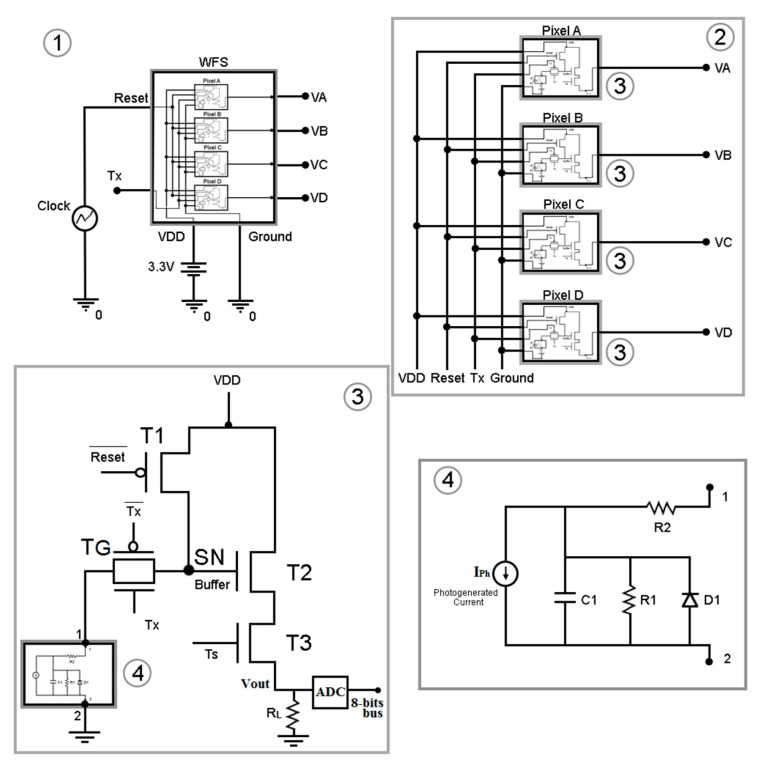
Schematics of the wavefront sensor (WFS) electronic interface: (**1**) main SPICE schematic; (**2**) quad-cell (QC) schematic, where VA is the output voltage level of pixel A, VB is the output voltage level of pixel B, VC is the output voltage level of pixel C and VD is the output voltage level of pixel D; (**3**) active-pixel sensor (APS) schematic, where VDD is the voltage source level; (**4**) photodiode schematic. T, transistor; SN, sense node; TG, transfer gate; R, resistor; C, capacitor; D, diode.

In Step 5, the wavefront is reconstructed, and there are two possible paths to go through the simulation. In Path 5.1, WF reconstruction uses the position coordinates from the spots as obtained in Step 4, enabling the testing of how much the QCs, microlenses and photodiodes individually impact the reconstruction. Choosing Path 5.2, the electronic structures are incorporated, allowing the evaluation of different pixel topologies and add-on circuitry prior to any fabrication step, either on-chip or on a printed-circuit board. In this path, the photocurrent signals obtained from Step 4 are transferred to the SPICE simulator, where a model was built that emulates the WFS electronic interface, as presented in Figure 3.

The top module in the SPICE schematics of Figure 3 is the WFS Model (1). The WFS is actually emulated, meaning that a single QC is simulated N times in a serial fashion, where N accounts for the number of microlenses in the array. This schematic diagram emulates the WFS behavior, producing a voltage for each pixel in a QC, resulting from the transducing of the input photocurrent in each of their respective photodiodes. The QC output voltages will then be employed to give information about the spot positions and further WF reconstruction, according to the model presented in [21]. Module (2) of Figure 3 represents each QC, composed by four pixels. In the real circuit layout, these pixels are symmetrically positioned about a common center and their photodetectors separated by a small gap, like presented in Figure 1e. Every pixel in the QC has the same circuit design, and their common structure is represented in Module (3). The employed circuit topology was the classical 5-transistor active-pixel sensor (5T APS) [27], composed by a photodiode, 2 PMOS and 3 NMOS transistors (sizing parameters in Table A1), whose function is to transduce the input photocurrent to an output voltage. Furthermore, an 8-bit analog-to-digital converter (ADC) is used to convert this signal into a digital format. Lastly, the equivalent circuit of the photodiode is shown in (4), which allows the simulation of the photodetector behavior in the SPICE environment [26].

All of the integrated components of the WFS that receive information about the photogenerated current ($I_{ph}$) from a file generated by the C algorithm were designed in a standard AMS CMOS 0.35-µm technology that uses reliable semi-empirical models for integrated devices’ simulation [28]. This photogenerated current is represented by a current-source in the photodiode Model (4) in Figure 3, which in turn is used inside the APS circuit (Module (3)).

The APS is composed of five transistors. The reset transistor (T1) works as a switch and when turned on is used to charge the photodetectors intrinsic capacitance, setting a reference voltage source level (VDD) at the sense node (SN). The buffer transistor (T2) buffers the signal to the selection transistor (T3), which is a switch that enables the selection of the pixel to be read. When turning off T1 and considering that the signal Tx at the transfer gate TG is on—where TG is a PMOS/NMOS complementary switch—the photodetector’s internal capacitance starts to discharge at a rate proportional to its photocurrent, reducing the voltage on the SN. Therefore, the voltage drop at the SN, which determines the output Vout, is a measure of the photocurrent’s intensity. When TG is turned off, the voltage at SN can be kept rather constant during a storage period for further readout purposes.

**Figure 4:**
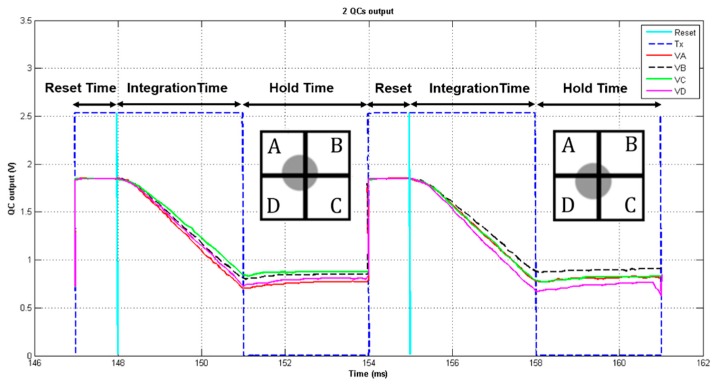
Voltage vs. time graph showing reset, Tx and output signals from two QCs read when Tx toggles to the off state, at 151 ms and 158 ms for the first and second QC, respectively.

Figure 4 shows a voltage vs. time graph, comprising the reset time, the integration time (when Tx is on), the hold time (when Tx is off) and the output signals the output voltage levels of each APS of the QC (VA, VB, VC, VD) of two QCs after going through the electronic-circuit path in SPICE. It also shows the relative position of the spot over each QC.

The read-out voltage for each QC is sampled just after Tx is turned off, for instance at 151 ms for the first QC in Figure 4, at 158 ms for the second QC, and so on. In practice, every analog signal is converted to a digital form through an ADC. In this work, an 8-bit integrated ADC SPICE model, optimized for the AMS 0.35-μm technology, was employed, with the reference voltage equal to VDD. The read-out output voltages, converted to their corresponding digital values, were then exported to the C algorithm as a text file to reconstruct the spot coordinates, where each signal is the reference reset voltage minus the voltage read by each pixel output.

The x and y spot-centroid coordinates enable the reconstruction of the wavefront. Either Step 5.2 or 5.1 of Figure 2 uses the same C algorithm to reconstruct the WF, which is done through the least-squares method, in order to find the best coefficients ($C_{out}$) of a weighted sum of Zernike polynomials, given the chosen number of Zernike base functions in Step 1 of Figure 2. Finally, in Step 6, the error in the reconstruction process, due to the whole sampling and measurement process, is estimated. The RMS (root-mean-square) reconstruction error calculation between the output wavefront, $W_{out}\left(x,y\right)$, and the original wavefront, $W_{in}\left(x,y\right)$, is given by:

(1) $$W_{rms}=\sqrt{\frac{\sum_{1}^{n}{\left(W_{out}\left(\xi_{n},\varphi_{n}\right)-W_{in}\left(\xi_{n},\varphi_{n}\right)\right)}^{2}}{n-1}},$$

where *n* is the total number of points in the sampling grid in which the WF function is described, and the values $W_{out}\left(x,y\right)$ and $W_{in}\left(x,y\right)$ are calculated for each point ($\xi_{n}$, $\varphi_{n}$) using Equations (2) and (3):

(2) $$W_{out}\left(\xi_{n},\varphi_{n}\right)=\overset{M}{\underset{k=0}{\sum }}C_{{out}_{k}}Z_{k}\left(\xi_{n},\varphi_{n}\right)$$

(3) $$W_{in}\left(\xi_{n},\varphi_{n}\right)=\overset{M}{\underset{k=0}{\sum }}C_{{in}_{k}}Z_{k}\left(\xi_{n},\varphi_{n}\right).$$

Both coefficients $C_{{out}_{k}}$ and $C_{{in}_{k}}$ represent the amplitudes of each Zernike function $Z_{k}$ composing the WF. Terms $C_{{in}_{k}}$ are the input coefficients according to Porter’s statistics [25], and $C_{{out}_{k}}$ are the obtained output coefficients through the WFS simulation platform.

## 3. Simulation Results

### 3.1. Considerations

The simulation procedure described in the previous section enables the design of optimal electronic and optical parameters, in order to suit different applications, before the sensor is actually manufactured. Besides contributing to sensible decisions about the sensor design, the simulation also allows one to assess the separate and combined impact of optical, optoelectronic and electronic parameters in the sensor performance. To exemplify the importance of the platform, we have varied some parameters, combined with the presence or absence of the electronic circuitry, in order to observe their impact on the overall sensor performance. The chosen parameters were the number of microlenses in the microlens array and the type of PSD quadrant detector (QC_H_ or QC_D_). All variables used in the simulations are shown in Table A1.

The number of microlenses, their distribution pattern and size have a direct influence on the WF sampling, promoting in some cases an increase in the reconstruction error [29]. The simulation of the electronic modules helps in the evaluation of how much error the read-out electronics introduce in the final wavefront reconstruction. As described earlier, the simulations were performed according to the device models of the AMS CMOS 0.35-μm technology, and although process variations were not yet considered in these simulations, it is possible to perform Monte Carlo analysis, emulating CMOS process parameters’ variation. Furthermore, the read-out process introduces noise, modelled by both SPICE and the C algorithm as a Gaussian noise distribution around the read voltage by the pixel. Noise was estimated in SPICE based on the power-spectral-density of the circuit. The integration of the power-spectral-density curve throughout the whole circuit bandwidth results in the RMS read-noise voltage. Frequency-domain SPICE simulations for several photocurrents were performed, each resulting in a different power-spectral-density, and the worst case was chosen, i.e., the one that yielded the largest RMS noise voltage. Then, the C algorithm uses that RMS voltage as the standard deviation σ for a random Gaussian additive noise generator, in order to account for the read-noise contribution. This noise estimation in the SPICE is necessary, since the time-domain simulations, as exemplified by Figure 4, are noiseless, always resulting in the same read-out voltage, for a given input photocurrent. Next, the C code uses the pixel read-out voltage value added by the random noise contribution as the final pixel output, used to further reconstruct the WF. Each pixel, for every input photocurrent, with its respective output read-noise contribution, was simulated 100 times, in order to achieve a large enough population for the WFS reconstruction, whose average and standard deviation were extracted, neglecting further optoelectronic noise components, like photodetector and photonic noise, which will not be contemplated in this work.

**Figure 5:**
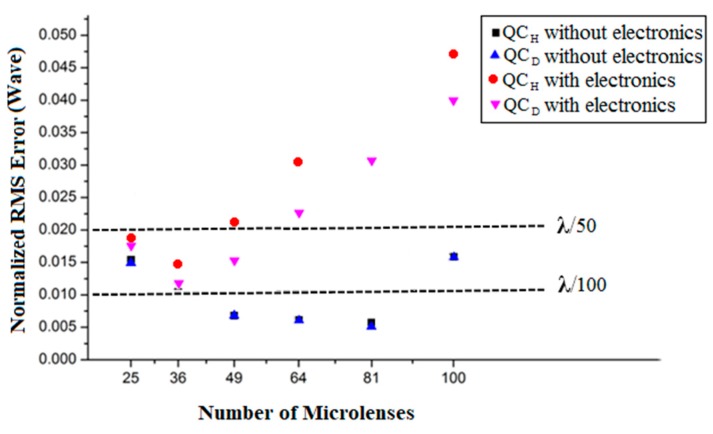
Normalized RMS error contribution of the microlenses, QC type and electronic modules (without ADC).

Twenty Zernike polynomials were used for both the WF generation and its reconstruction, not considering piston, tilt x, tilt y and defocus. The terms piston and tilt were discarded from the reconstruction for not being relevant to ophthalmological aberrations, and the defocus term was not considered due to its relatively high coefficient, which would dominate the wavefront reconstruction, obliterating the analysis of finer terms. The average coefficient values for ocular aberrations of a population of individuals, registered by Porter et al. [25], describe the deployed input wavefront. In the results displayed in the simulations of Figure 5, the minimum number of microlenses considered in an orthogonal array was 25, in order to minimize the sampling error, assuring a reliable WF reconstruction by having enough sampling points available to represent the targeted Zernike reconstruction modes [30]. Further simulations with only 16 microlenses led to undersampling, and therefore to a significantly high reconstruction error and a less reliable reconstructed WF.

### 3.2. Results

Figure 5 shows the results of the RMS reconstruction error normalized by the wavelength (λ = 0.633 µm), for different numbers of microlenses and for both QC_H_ and QC_D_ quad-cell types. Furthermore, the increase of the normalized RMS error introduced by the read-out electronics can be observed. This is highlighted in Table 1, to evidence the significant difference between the reconstruction error with the addition of the pixel electronics relative to the error without it. The average comparison error $\bar{\Delta e}\;\left(\%\right)$ is calculated as follows:

(4) $$\bar{\Delta e}\;\left(\%\right)=\left(\frac{{\bar{\varepsilon }}_{EL}-{\bar{\varepsilon }}_{o}}{{\bar{\varepsilon }}_{o}}\right)\times 100,$$

where ${\bar{\varepsilon }}_{EL}$ is the average value of the RMS error with electronics and ${\bar{\varepsilon }}_{o}$ is the average value of the error without electronics.

Table 1 shows the relative percentage error over the influence of the electronic modules in the QCs.

At first, the results without the influence of electronic modules will be discussed. In Figure 5, it can be seen that, for arrays with microlenses between 25 and 81, the reconstruction error decreases as the number of microlenses increases, for both QC_H_ and QC_D_. This is due to the increasing number of sampling points, so the reconstruction will recreate the WF with higher fidelity. With 100 microlenses, however, the reconstruction error increased almost three times with respect to the error with 81 microlenses. In this case, the increase in the number of microlenses also reflects in more terms in the WF reconstruction matrix. It is important to note that besides specifying a number of microlenses that is suitable for sampling, it is desirable to have a number not too high, in order to keep the reconstruction error low. The maximum error depends on the application; for instance, for interferometry, it should be no more than λ/100; while for precision optical components, it should be lower than λ/50 [31].

In the simulations that use the electronic circuitry, the effects of different QCs turn out to be much more evident, as the QC_D_ shows a statistically-significant better performance between 25 and 81 microlenses, as presented in Figure 5, yielding smaller errors. Another important observation is that the larger the number of microlenses, the bigger the reconstruction error for 36 microlenses onwards. This makes sense, as more sources of numerical errors are being brought into the reconstruction algorithm, be it from the electronic devices, e.g., due to the APS transfer function, or from the voltage read-out noise. In Table 1, there is a quantitative view of the influence of electronics in the chosen structures analyzed. Every simulation presented in Figure 5 that follows Step 5.2 of Figure 2 (with electronics), instead of Step 5.1 (without electronics), shows a significant error increase. As an example, consider the case with 81 microlenses, as highlighted in Table 1. In this case, both QC_H_ and QC_D_ without electronics have the lowest absolute reconstruction RMS error. However, when the electronic modules are introduced, their RMS errors increase considerably. They show the largest relative percentage error of Table 1, the error with the electronics (besides that of the optical structures) approximately being 5.7-times larger for the QC_H_, and almost five times larger for the QC_D_. This indicates strongly that the analysis of electronic modules in the scope of the computational platform is of extreme importance to guide decisions concerning the sensor design. Another point to be taken into account with the effects of the electronics is the customization of the dimensions of the sensor in order to achieve the lowest reconstruction error. The customization of the sensor dimensions changes whether simulating with or without electronics. While without electronics, the smallest error is between 64 and 81 microlenses, with electronics, the number changes to nearly 36 microlenses. This influences a decision considering the WF sampling and the choice of the electronic devices, which also bring in noise sources. In Figure 5, it is shown that a better way to reconstruct a wavefront with high reliability and small reconstruction error using electronic modules is when 36 microlenses are employed. It is also worth noting that even when the electronic modules are employed, it is possible to reduce the reconstruction error, by properly designing a special QC, being in this case the QC_D_, which in turn is based on a specially-tailored photodetector structure, but compliant with standard CMOS technologies. Such additional error was observed for all the cases employing the electronic modules, but it appears that the difference between them is statistically insignificant for a number of microlenses higher than 81, due to read-noise.

**Figure 6:**
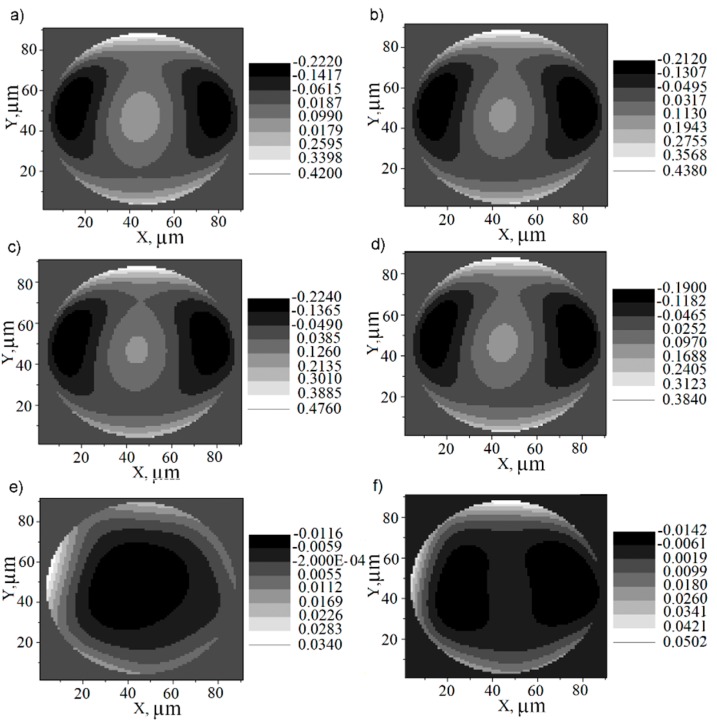
Average wavefront reconstructed from 100 simulation samples using 36 microlenses and homogeneous quad-cells with their respective RMS: (**a**) original WF; (**b**) reconstructed WF without electronics showing RMS error of $\bar{\varepsilon }=0.0104$ waves; (**c**) reconstructed WF with electronics showing $\bar{\varepsilon }=0.0152$ waves; (**d**) reconstructed WF with electronics plus an 8-bit ADC showing $\bar{\varepsilon }=0.0184$ waves; (**e**) residual WF (relative to the original one) without electronic modules; (**f**) residual WF (relative to the original one) with electronic modules.

Figure 6 shows a spatial distribution of the wavefronts (a) original, (b) reconstructed without electronic modules, (c) with electronic modules and (d) with electronics plus ADC, for a configuration of 36 microlenses of the QC_H_ type and their equivalent residuals, respectively in (e) and (f). The residual WF is the difference between the original WF and its reconstruction. Analysis of the residual values also helps in this evaluation, making the effect over the reconstruction RMS error more evident considering the electronic modules. The residual plots also show that the error along the borders is larger than that in the middle region of the WF, as expected since the wavefront aperture circumscribes the orthogonal microlens array area. However, when the signal passes through the electronic circuitry, the error is more pronounced in the central regions of the WF, where the tilts of the surface patches are smaller, and their respective light spots fall about the center of the QCs where it features a more linear response. In this case, the reconstructed spot positions are more susceptible to read-out noise of each of the four pixels in the QC, yielding a slightly larger reconstruction error. Moreover, the presented RMS error reconstruction in Figure 6 is considered to have acceptable values in the context of ophthalmology. When a WF is reconstructed without integrated electronics, it presents an RMS error around λ/100; with integrated electronics the value changes to λ/70; and when the 8-bit ADC is introduced, the error rises to λ/50, which nevertheless still proves acceptable.

## 4. Conclusions

The simulations performed for a Hartmann-Shack WFS including integrated electronics serve as an advantageous auxiliary tool in designing the sensor, especially when a custom focal-plane chip is intended. The extension of the simulation to include electronics enables the performance assessment of different types of read-out circuits, position-sensitive detectors and imaging arrays prior to their manufacturing. It is important to observe that the RMS reconstruction error increases considerably when the electronic modules are taken into account, affecting the signals that are used by the reconstruction algorithm. The second important observation is that, even when the electronic modules are employed, it is possible to reduce the reconstruction error by properly designing the position-sensitive detector, being in this case a quad-cell with a double efficiency concentric structure (QCD). In addition, combining reliable integrated circuit simulation or that of discrete electronic components, to existing and more ample adaptive optics simulation platforms will not only contribute to enhancing the reconstruction fidelity featured by wavefront sensors, but also to the assessment of the complete adaptive optics system.

## Figures and Tables

**Table 1:** Comparison error (%) using electronics relative to without electronics.

Number of Microlenses	${QC}_{H}\;\bar{\Delta e}\;\left(\%\right)$	${QC}_{D}\;\bar{\Delta e}\;\left(\%\right)$
25	17	15
36	41	12
49	211	122
64	392	271
81	571	504
100	197	153

## Appendix A

**Table A1:** Initialization parameters from the computational platform.

Symbol	Parameter	Simulation Value	Unit	Step
Λ	Operational wavelength	0.633	µm	1
	Number of Zernike polynomial terms	20	-	1
	Quantity of microlenses in the microlens array	25, 36, 49, 64, 81, 100	-	3
D_microlens_	Diameter of each microlens	1000	µm	3
	Lateral dimension of the microlens array	6000	µm	3
F	Focal distance	200,000	µm	3
R_QC_	Dimension of a cell from the QC	200	µm	4
	Spacing between QCs	10	µm	4
r_c_	Central radius of the QC_D_	0.325 R_QC_	µm	4
η_r_	Relative quantum efficiency of the QC_D_	0.667	-	4
R_eff_	Spot effective radius	0.30 R_QC_	µm	4
	Linearization	0.46 R_QC_	µm	4
α_m_	Absorption coefficient of the material (Si)	3345	cm^−1^	4
R	Reflectance	0.1		4
L_n_	Diffusion length of electrons	87.68 × 10^−6^	cm	5
L_p_	Diffusion length of holes	29.30 × 10^−3^	cm	5
D_n_	Diffusivity of electrons	2.23	cm^2^/s	5
D_p_	Diffusivity of holes	10.76	cm^2^/s	5
S_N_	Surface recombination speed for carriers in the cathode	70	cm/s	5
S_P_	Surface recombination speed for carriers in the anode	4000	cm/s	5
Cj	Capacitance of the photodiode junction	2	pF	5

The physical parameters for the electronic components were those for the standard AMS CMOS process, label 0.35 µm.
